# Supplementary material for: Acinetobacter spp. with lower susceptibility to quaternary ammonium compounds enriched in microbial communities of frequently used sinks
Source: Appl Environ Microbiol. 2026 Mar 17;92(4):e01968-25. doi: 10.1128/aem.01968-25 (PMC13101483; doi:10.1128/aem.01968-25)
Supplement: Supplemental material — Tables S1 to S5; Fig. S1 to S10. [file aem.01968-25-s0001.pdf]

Supplementary information

Table S1. Number of biofilm samples collected in each Building

Buildings	Use	Age	Sampling points	#samples
EH	Academic	New (11)	Restroom L1 (Sinks A and B)<br>Restroom L2 (Sinks A and B)<br>Restroom L3 (Sinks A and B)<br>Restroom L4 (Sinks A and B)	144
WC		Old (62)	Restroom L1 (Sinks A and B)<br>Restroom L2 (Sinks A and B)<br>Restroom L3 (Sinks A and B)<br>Restroom LB (Sinks A and B)	144

Season	Traffic	Time-points
Winter	Low (Jan-20)<br>High (Jan-28)	Pre-AM<br>Post-AM<br>PM
Spring	High (March-10)<br>Low (March-17)	
Summer	Low (Aug-22)<br>High (Sept-6)	

Table S2. Building's people traffic (summer season)

**Building**	**WC**								**EH**							
**Floor**	Basement (LB)		First (L1)		Second (L2)		Third (L3)		First (L1)		Second (L2)		Third (L3)		Fourth (L4)	
**Traffic**	Low	High	Low	High	Low	High	Low	High	Low	High	Low	High	Low	High	Low	High
**Counts (average/day)**	**2.3**	**5.8**	**30.8**	**45**	**13**	**16**	**12**	**14**	**9.33**	**33.5**	**10**	**25**	**12.8**	**10**	**4.75**	**14**

*average four consecutive days

Table S3. Chemical characterization of bulk water samples

Building	Campaign	Time	Be	Na	Mg	Al	K	Ca	V	Cr	Mn	Fe	Co	Ni	Cu	Zn	As	Se	Mo	Ag	Cd	Hg	Tl	Pb
EH	W-LT	PM	0.01	12.83	12.65	<0.000	0.92	38.49	0.25	0.37	1.45	0.02	0.08	6.53	136.40	353.74	0.71	0.41	0.74	<0.000	0.05	0.13	0.01	0.00
EH	W-HT	AM	0.01	13.66	13.09	<0.000	0.88	38.20	0.34	0.26	0.56	0.00	0.05	3.56	204.23	178.28	0.52	0.30	0.65	<0.000	0.01	0.05	0.00	0.05
EH	W-HT	PM	0.00	13.43	13.06	<0.000	0.92	38.40	0.31	0.29	0.50	0.00	0.03	3.00	159.21	131.63	0.55	0.09	0.67	<0.000	0.01	0.03	0.00	<0.000
EH	Sp-HT	AM	<0.000	28.49	12.12	17.11	0.88	32.31	0.28	0.30	0.48	0.00	0.03	1.33	312.98	7.51	0.34	0.46	0.45	0.98	0.00	0.00	0.00	0.44
EH	Sp-HT	PM	<0.000	10.08	12.59	3.22	0.95	36.33	0.26	0.20	0.39	0.00	0.04	2.41	67.97	198.34	0.38	0.17	0.48	0.01	<0.000	0.05	0.00	0.01
EH	Sp-LT	AM	0.00	10.05	12.91	<0.000	1.24	37.63	0.32	0.32	0.58	0.00	0.05	7.97	262.65	834.41	0.53	0.37	0.66	<0.000	0.00	0.04	0.00	<0.000
EH	Sp-LT	PM	0.00	10.44	13.15	<0.000	1.28	37.94	0.61	13.34	3.22	0.53	0.15	2.90	243.46	238.94	0.59	0.43	4.52	<0.000	0.00	0.05	0.00	<0.000
EH	Su-LT	AM	0.00	10.43	12.50	19.73	0.91	35.65	0.29	0.26	0.43	0.01	0.04	1.22	268.99	39.53	0.57	0.18	0.45	0.01	<0.000	0.02	0.00	<0.000
EH	Su-LT	PM	0.01	10.53	12.65	11.78	0.96	36.96	0.25	0.24	0.60	0.01	0.05	1.51	100.84	65.64	0.55	0.17	0.43	<0.000	<0.000	0.02	0.00	<0.000
EH	Su-HT	AM	0.00	10.47	12.72	17.70	0.91	36.08	0.26	0.22	0.52	0.00	0.02	4.06	103.62	508.25	0.56	0.33	0.44	<0.000	<0.000	0.01	0.00	<0.000
EH	Su-HT	PM	0.03	10.71	12.67	28.10	0.94	35.78	0.26	0.23	0.50	0.00	0.02	1.89	95.88	152.63	0.62	0.18	0.44	<0.000	<0.000	0.00	0.00	<0.000
WC	W-LT	PM	0.00	13.07	12.68	<0.000	0.93	38.23	0.27	0.80	0.74	0.01	0.04	2.21	398.66	131.00	0.48	0.23	0.66	<0.000	<0.000	0.10	0.01	<0.000
WC	W-HT	PM	0.01	13.85	13.13	<0.000	0.87	38.28	0.32	0.30	0.82	0.00	0.05	2.00	162.71	161.17	0.55	0.38	0.62	<0.000	0.01	0.02	0.00	0.01
WC	W-HT	AM	0.00	13.78	13.06	<0.000	0.83	41.79	0.34	0.25	1.34	0.00	0.06	1.40	137.45	348.95	0.52	0.22	0.67	<0.000	0.02	0.03	0.00	0.03
WC	Sp-HT	PM	0.03	30.09	12.93	22.57	1.26	37.43	0.31	0.33	0.76	0.00	0.05	0.99	131.36	29.97	0.53	0.60	0.64	0.30	0.00	0.24	0.00	0.03
WC	Sp-HT	AM	0.00	28.90	12.89	<0.000	1.25	37.16	0.33	0.32	0.69	0.00	0.05	0.96	137.82	17.91	0.54	0.47	0.62	0.32	<0.000	0.07	0.00	0.05
WC	Sp-LT	PM	0.01	31.44	12.89	<0.000	1.25	37.44	0.32	0.49	0.81	0.00	0.03	1.58	119.21	53.70	0.65	0.48	0.65	0.17	0.02	0.04	0.00	0.02
WC	Sp-LT	AM	0.01	31.16	12.70	<0.000	1.22	36.47	0.29	0.31	1.02	0.00	0.05	0.92	121.34	50.09	0.68	0.52	0.64	0.29	0.01	0.01	0.00	0.01
WC	Su-LT	AM	<0.000	10.78	12.57	25.77	0.92	36.02	0.29	0.24	1.36	0.01	0.03	0.47	32.66	29.78	0.59	0.22	0.45	0.08	<0.000	0.01	0.00	<0.000
WC	Su-LT	PM	0.03	10.41	12.55	31.58	0.92	35.46	0.28	0.22	1.28	0.00	0.01	0.30	24.92	16.92	0.60	0.17	0.42	0.04	<0.000	0.01	0.00	<0.000
WC	Su-HT	AM	0.03	10.89	12.68	26.24	0.96	36.10	0.28	0.22	0.80	0.00	0.02	0.57	49.69	33.38	0.64	0.15	0.48	<0.000	<0.000	<0.000	0.00	<0.000
WC	Su-HT	PM	0.00	10.59	12.62	54.11	0.93	35.97	0.28	0.22	0.67	0.00	0.02	0.22	24.86	11.41	0.60	0.13	0.46	<0.000	<0.000	<0.000	0.00	<0.000

Note1: All units are in ppb. Campaigns: W-LT (Winter-low traffic), W-HT (Winter High Traffic), Sp-LT (Spring Low traffic), Sp-HT (Spring High-traffic), Su-LT (Summer, Low-traffic); Su-HT (Summer, High-traffic).

Note2: Composite sink water samples were collected during all sampling campaigns. The two missing entries correspond to the winter low-traffic morning (AM) campaign, where only samples for DNA extraction were collected.

Table S4. Characterization of high-quality Metagenome-Assembled Genomes (HQ-MAGs) based on MIMAG criteria as calculated by CheckM from directly sampled sink drain swabs.

Building	Bin_ID	Taxa (gtdbtk)	Completeness (%)	Contamination (%)	Genome size (bp)	N50 (scaffolds)	Mean scaffold length (bp)	Longest scaffold (bp)	GC (%)	ANI to closest
EH	**bin_S1_1**	*Proteobacteria;c_Gammaproteobacteria;o_Burkholderiales;f_Burkholderiaceae; g_Ottowia;s_Ottowia sp018060485*	99.53	2.23	4073937	27295	12612	109854	66.40	98.89
	**bin_S1_2**	*Actinobacteriota;c_Actinomycetia;o_Mycobacteriales;f_Nakamurellaceae; g_Nakamurella;s_N. flava*	99.23	1.37	4928184	127987	57978	368635	71.38	85.42
	**bin_S2_1**	*Actinobacteriota;c_Actinomycetia;o_Mycobacteriales;f_Mycobacteriaceae; g_Mycobacterium;s_M. phocaicum*	97.36	5.01	7216698	19993	10707	293021	66.37	95.30
	**bin_S2_2**	*Proteobacteria;c_Alphaproteobacteria;o_Rhodobacterales;f_Rhodobacteraceae ; g_Amaricoccus*	98.40	2.80	3740913	29492	15918	117752	70.35	NA
WC	**bin_S3_1**	*Actinobacteriota;c_Actinomycetia;o_Mycobacteriales;f_Mycobacteriaceae; g_Mycobacterium*	96.42	4.47	7753530	118794	34307	374098	65.50	NA
	**bin_S4_5**	*Proteobacteria;c_Gammaproteobacteria;o_Pseudomonadales;f_Moraxellaceae; g_Acinetobacter;s_A. parvus*	99.18	3.35	2747045	20116	11446	56461	41.93	96.42
	**bin_S4_9**	*Bacteroidota;c_Bacteroidia;o_Flavobacteriales;f_Weeksellaceae; g_Chryseobacterium;s_Chryseobacterium sp902506175*	98.52	3.25	5457457	47674	12545	185203	33.33	99.12

Table S5.1. MAGs recovered from BAC-containing plates*. Presence of species from directly sampled sink drain swabs.

Taxa	Swab	R2A	BAC1	BAC10	BAC100	V1	V3
Pseudomonadota;c__Gammaproteobacteria;o__Burkholderiales;f__Burkholderiaceae;g__Ottowia;s__Ottowia sp018060485	Present ■	Absent	Absent	Absent	Present ■	Present ■	Absent
Pseudomonadota;c__Gammaproteobacteria;o__Pseudomonadales;f__Moraxellaceae;g__Acinetobacter;s__Acinetobacter parvus	Present ■	Absent	Present	Present ■	Present	Present ■	Present
Pseudomonadota;c__Gammaproteobacteria;o__Pseudomonadales;f__Moraxellaceae;g__Moraxella_A;s__Moraxella_A sp902506215	Present ■	Absent	Absent	Absent	Present ■	Present	Absent
Bacteroidota;c__Bacteroidia;o__Flavobacteriales;f__Weeksellaceae;g__Chryseobacterium;s__Chryseobacterium sp902506175	Present ■	Present	Present ■	Present	Absent	Absent	Absent

Present
Absent
■ HQ/MQ MAG

Table S5.2. MAGs recovered from BAC-containing plates*. Presence of species abundant on the 16S dataset.

Taxa	16 S (%)	R2A	BAC1	BAC10	BAC100	V1	V3
Bacteroidota;c__Bacteroidia;o__Flavobacteriales;f__Weeksellaceae;g__Chryseobacterium;s__Chryseobacterium formosense	1.2	Present	Present ■	Present ■	Present ■	Present ■	Absent
Bacteroidota;c__Bacteroidia;o__Flavobacteriales;f__Weeksellaceae;g__Chryseobacterium;s__Chryseobacterium aquaticum_A		Present ■	Absent	Present ■	Absent	Present ■	Absent
Bacteroidota;c__Bacteroidia;o__Flavobacteriales;f__Weeksellaceae;g__Chryseobacterium;s__Chryseobacterium mucoviscidosis		Present ■	Present ■	Present ■	Present ■	Present ■	Absent
Pseudomonadota;c__Alphaproteobacteria;o__Sphingomonadales;f__Sphingomonadaceae;g__Sphingomonas;s__Sphingomonas adhaesiva	13	Absent	Absent	Absent	Present ■	Present ■	Absent
Pseudomonadota;c__Alphaproteobacteria;o__Sphingomonadales;f__Sphingomonadaceae;g__Sphingomonas;s__Sphingomonas hankookensis_A		Present	Present ■	Present ■	Present ■	Absent	Absent
Pseudomonadota;c__Alphaproteobacteria;o__Caulobacterales;f__Caulobacteraceae;g__Brevundimonas;s__Brevundimonas diminuta_C	1.6	Absent	Absent	Present	Present ■	Present ■	Absent
Pseudomonadota;c__Alphaproteobacteria;o__Caulobacterales;f__Caulobacteraceae;g__Brevundimonas;s__Brevundimonas colombiensis		Present	Present	Present ■	Present ■	Present	Present
Pseudomonadota;c__Alphaproteobacteria;o__Caulobacterales;f__Caulobacteraceae;g__Brevundimonas;s__Brevundimonas huaxiensis		Absent	Absent	Present ■	Present	Present ■	Absent
Pseudomonadota;c__Alphaproteobacteria;o__Caulobacterales;f__Caulobacteraceae;g__Brevundimonas;s__Brevundimonas pondensis		Present	Present ■	Present ■	Absent	Present	Absent

Present
Absent
■ HQ/MQ MAG

*Varying concentrations of BAC (C_{14} 50%, C_{12} 40%, C_{16} 10%) at 1 (BAC1), 10 (BAC10), and 100 (BAC100) mg/L, and commercial disinfectant Virex®Plus, tested at two dilutions: V1 (1:16,000 times), corresponding to a BAC concentration of 5.4 mg/L, and V3 (1:2,000 times - 8 times more diluted than recommended), corresponding to 43.5 mg/L BAC. The commercial disinfectant formulation included BAC (8.9%) and other QAC biocides: 6.67% octyl decyl dimethyl ammonium chloride, 2.67% dioctyl dimethyl ammonium chloride, and 4% dodecyl dimethyl ammonium chloride.

Drain Biofilm Sampling Procedure

Biofilm samples were collected from the inner sink drains, specifically from the are beneath the metal drain strainer, which is not reached during routine surface cleaning. The metal strainer remained in place throughout the procedure to ensure sampling of the biofilm as it naturally develops. A sterile 10-cm swab was inserted approximately 5 cm below the sink strainer through two opposite perforations and rotated to scrape the biofilm layer along the drain walls for 5 seconds (Figures SA1-A,B). This approach targeted the upper biofilm, a region where residues from daily use and intermittent disinfectant droplets accumulate. To obtain representative coverage, the swab was inserted sequentially into two opposite accessible perforations of the drain strainer. The researcher wore clean nitrile gloves and avoided any contact with surrounding upper surfaces to prevent contamination. After sampling, the swab was placed into a commercial sterile tube containing a buffer solution and immediately transported on ice to the laboratory for processing.

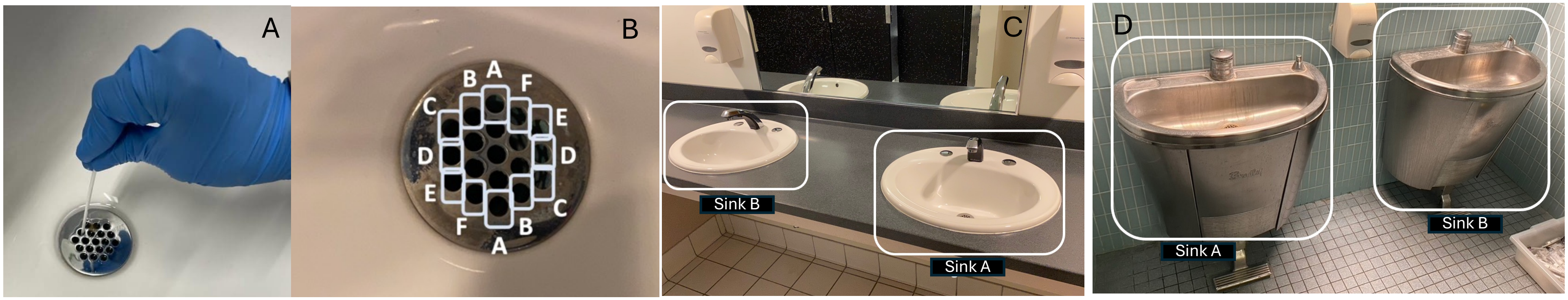

Fig. SA1. Drain Biofilm Sampling Procedure. A. Sampling of sink drain biofilm. A sterile 10-cm swab was inserted approximately 5 cm beneath the metal drain strainer through one of the perforations and rotated to collect biofilm from the interior drain walls. The metal strainer remained in place during sampling, and the sampler wore clean nitrile gloves to avoid contamination. **B**. Schematic representation of the drain strainer perforations sampled across time points and seasons. During the low-traffic period, opposing perforations were sampled in pairs (A–A, B–B, C–C): A–A in the morning before the first flush, B–B immediately after the first flush, and C–C in the afternoon (pm). During the high-traffic season, additional perforation pairs (D, E–F) were sampled to capture increased usage patterns. Each sampling event used a new sterile swab inserted through both perforations of the designated pair. **C**. Ceramic drop-in sinks in the first and second floor women's restroom of the Wehr Chemistry (WC) building (similar sinks are in EH). Both sinks are mounted in a solid-surface countertop and equipped with standard metal drain strainers. **D.** Stainless-steel wall-mounted utility sinks (Bradley-style) in the basement and third floor women's restroom of the WC building. These high-traffic fixtures include integrated stainless-steel basins and metal drain strainers.

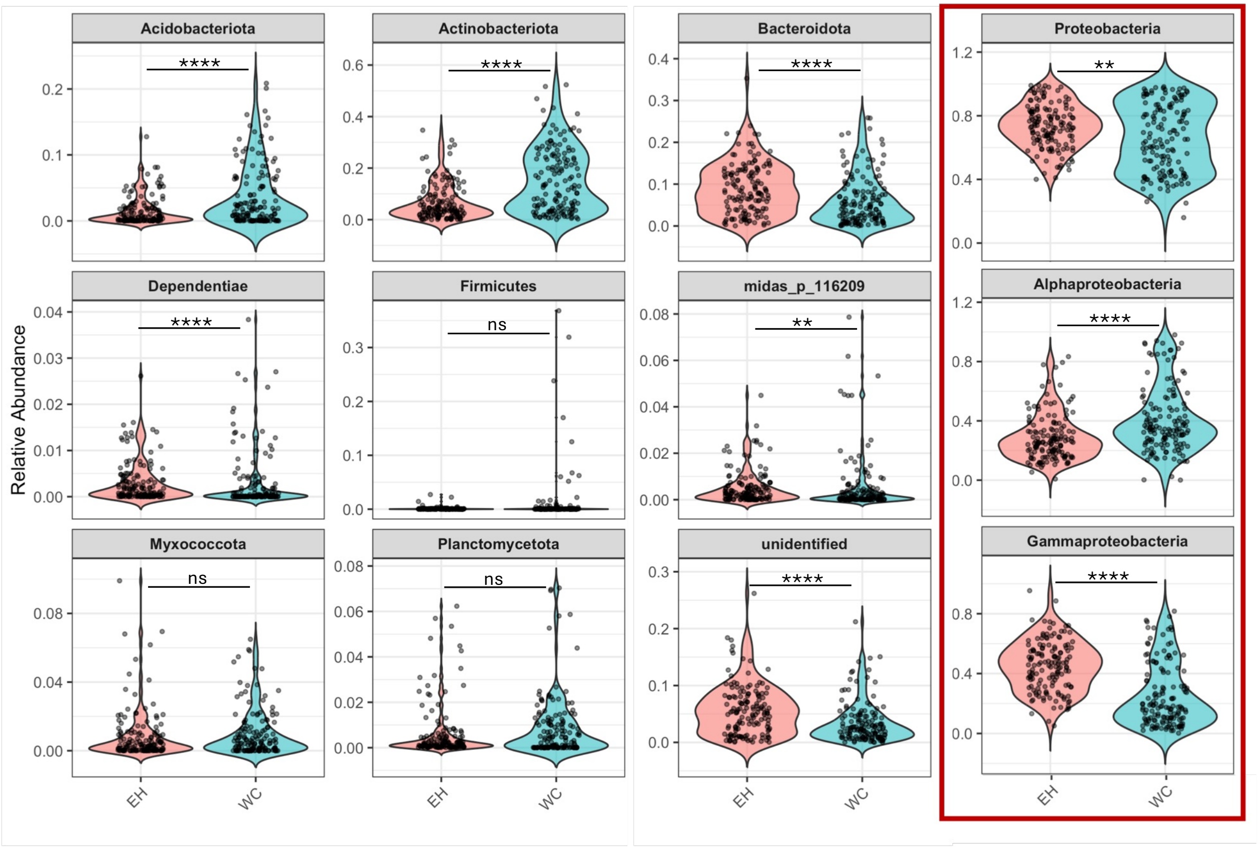

Fig. S1. Microbial community. Top ten phyla and key Proteobacterial classes by building. Differences were tested by Wilcoxon test **** *p < 0.0001; *** p < 0.001; ** p < 0.01; * p < 0.05* and 'ns' non-significant

Similar to the findings of Withey et al. (2021) and Hu et al. (2023), who explored microbial communities in communal and dormitory university sinks, our analysis revealed *Proteobacteria* as the dominant phylum, accounting for 73.6 % in EH and 65.6% in WC. Within this phylum, the class *Gammaproteobacteria* was significantly more abundant in EH (44.7%) compared to WC (25.2%). Conversely, class *Alphaproteobacteria* was more prevalent in WC (40.4%) than in EH (28.8%). *Actinobacteriota* and *Firmicutes* were also among the most abundant phyla, contrasting with the EH building, where *Bacteroidota* was the second most abundant phylum following by *Actinobacteriota and Acidobacteriota*. Our findings indicate that Gram-positive bacteria were more prevalent in the older building which contrasts with the results of Mahnert et al (2019), who showed that increased confinement and frequent cleaning of surfaces are associated with a shift from Gram-positive bacteria, such as *Actinobacteria* and *Firmicutes*, to Gram-negative such as *Proteobacteria* (Mahnert et al., 2019). This suggests that environmental conditions specific to sink drains may play a key role in shaping the microbial community. Withey and Gweon (2024) conducted a longitudinal study over 2.5 years on bacterial community dynamics of the sink microbiome in a newly built university building and found that after 98 weeks, *Proteobacteria* and *Bacteroidota* were predominant (71.2% and 27.3%, respectively).Notably, the differences in the relative abundances of *Gammaproteobacteria* and *Alphaproteobacteria* between EH and WC in our study may be attributed to factors such as building age, sink design, and user behavior influencing the structure of microbial communities.

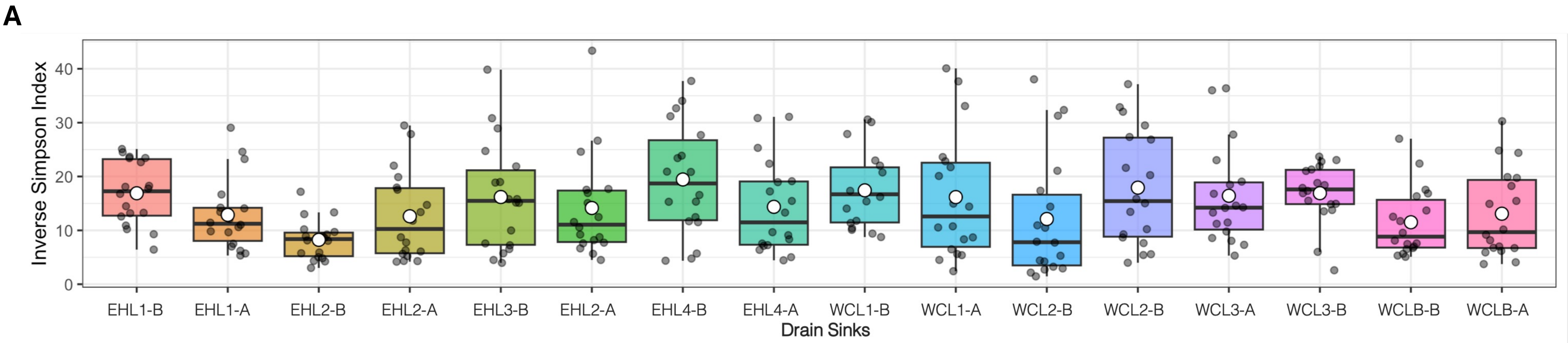

Fig S2.A. Alpha diversity (measured with Inverse Simpson diversity). Boxes depict the median and first and third quartiles. Whisker lines extend to interquartile ranges × 1.5 and points are outlier values (n=18/sink). Significant differences were found between sinks B of floors EHL2 vs EHL1 and EHL4.

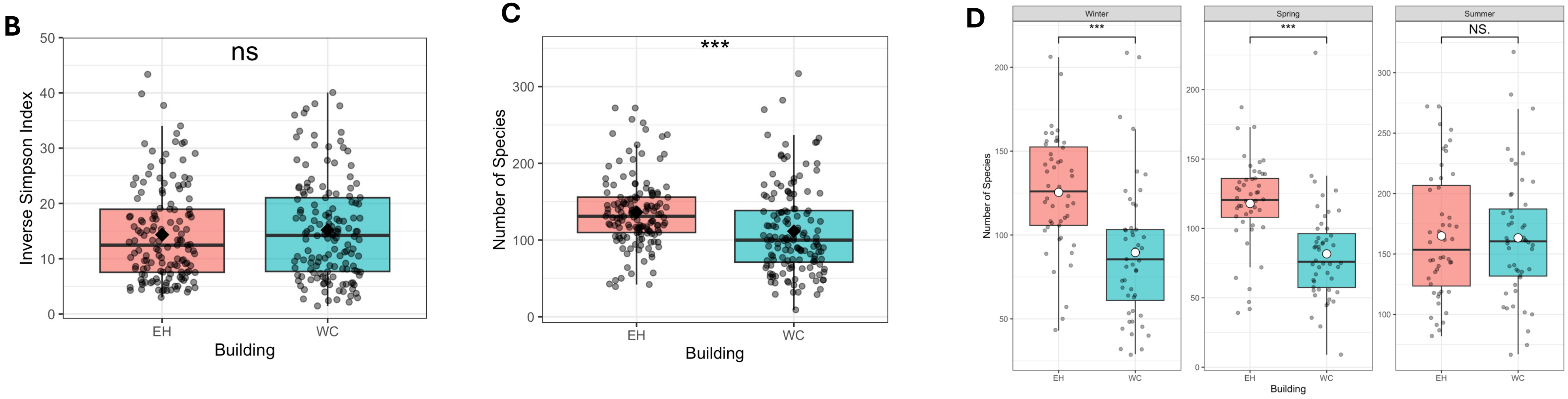

Fig S2.B. Alpha diversity indexes by building (measured with Inverse Simpson diversity). C. Number of species per building. D. Number of species per building by season Boxes depict the median and first and third quartiles. Whisker lines extend to interquartile ranges × 1.5 and points are outlier values.

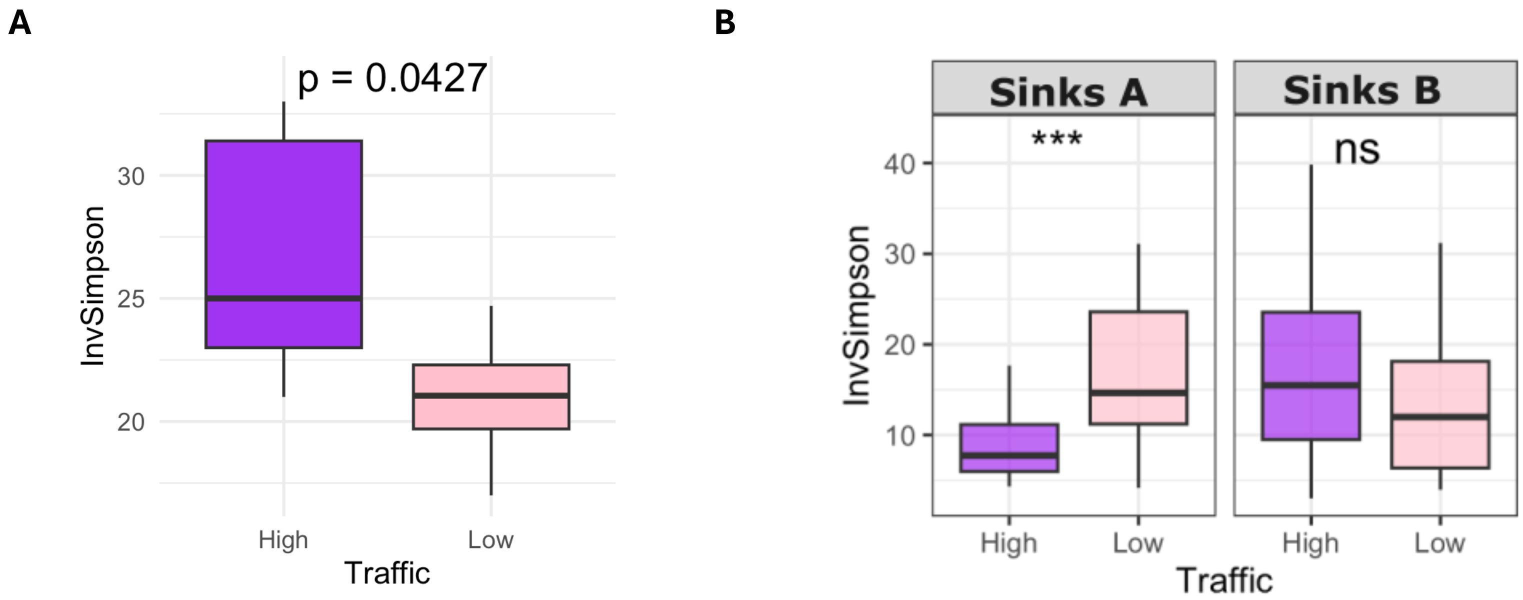

Fig. S3. Alpha diversity during periods of low and high traffic in the EH building. **A.** Water phase (composite sample). **B.** Biofilm phase, by traffic and location of sink (A: nearest to the door; B: furthest to the door). Sinks A experienced higher usage patterns.

In the EH building, the InvSimpson alpha-diversity index of water samples, increased from winter (19.0) to spring (22.4) and summer (29.7). The diversity was also statistically higher when more people were present in the building compared to periods of low traffic ($p < 0.043$).

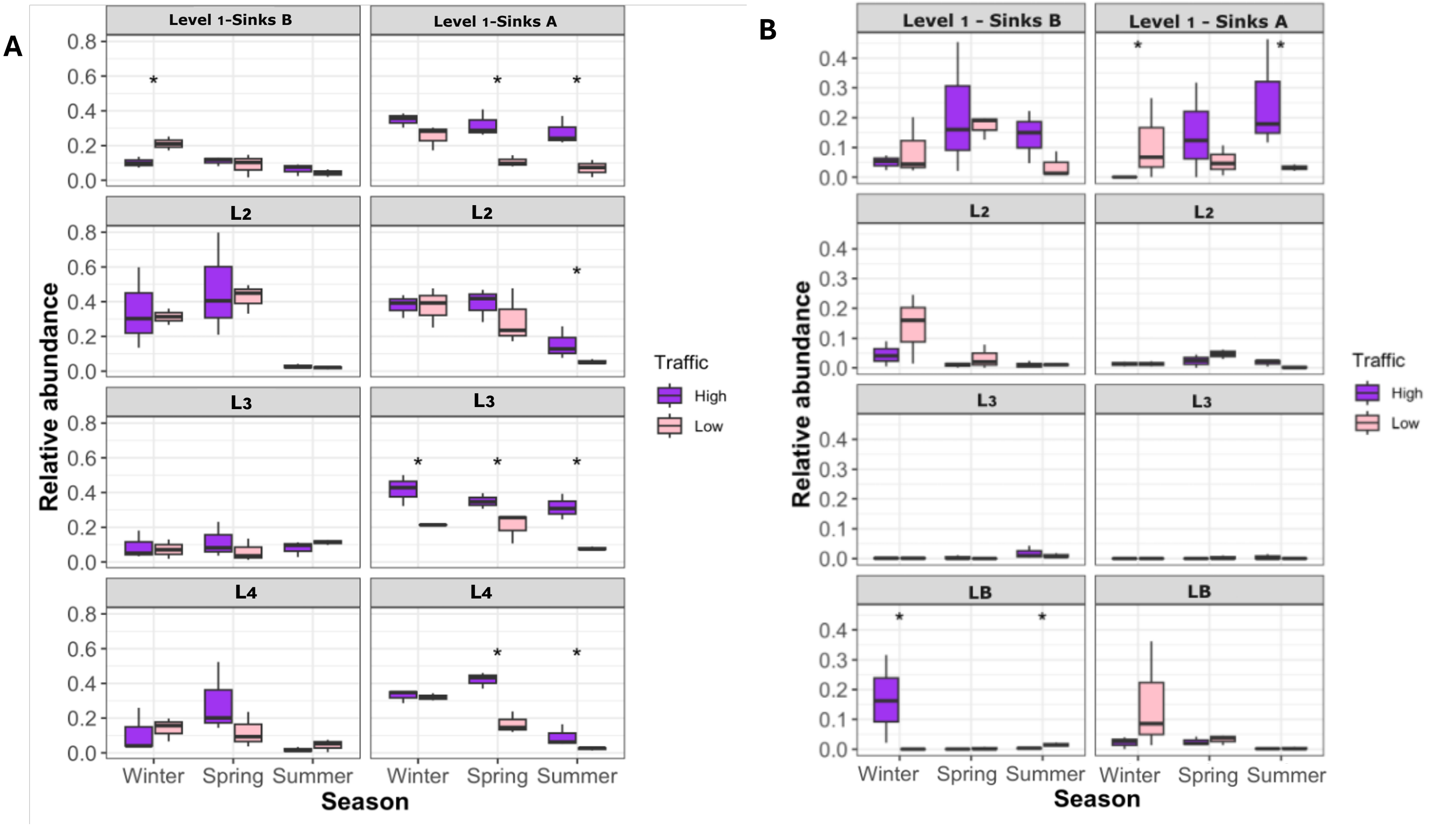

Fig. S4. Relative abundance of genus *Acinetobacter* in sinks A and B biofilms located in EH (A) and WC (B) by floor (Level), sampled during periods of high (HT, purple) and low traffic (LT, pink) of people at three different seasons.

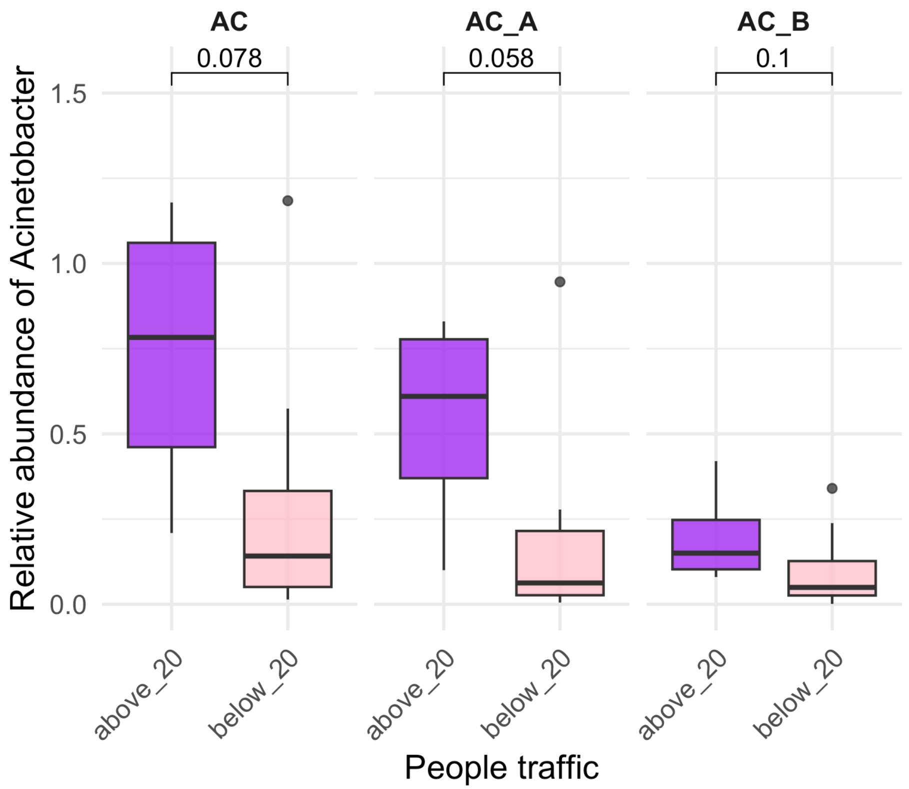

Fig. S5. Relative abundance of genus *Acinetobacter* by floors with high traffic (above 20 occupants) and low traffic (below 20 occupants) during the summer season. “AC_A" and ”AC_B" represent *Acinetobacter* abundance in samples collected from the sinks located closest and furthest to the entrance, respectively; “AC” represent the total abundance of *Acinetobater* (A + B) per floor. Wilcoxon rank-sum tests showed no statistically significant differences between traffic groups for any of the sink positions (AC_A: $p = 0.058$; AC_B: $p = 0.103$), although a trend toward higher abundance in high-traffic floors was observed in AC_A.

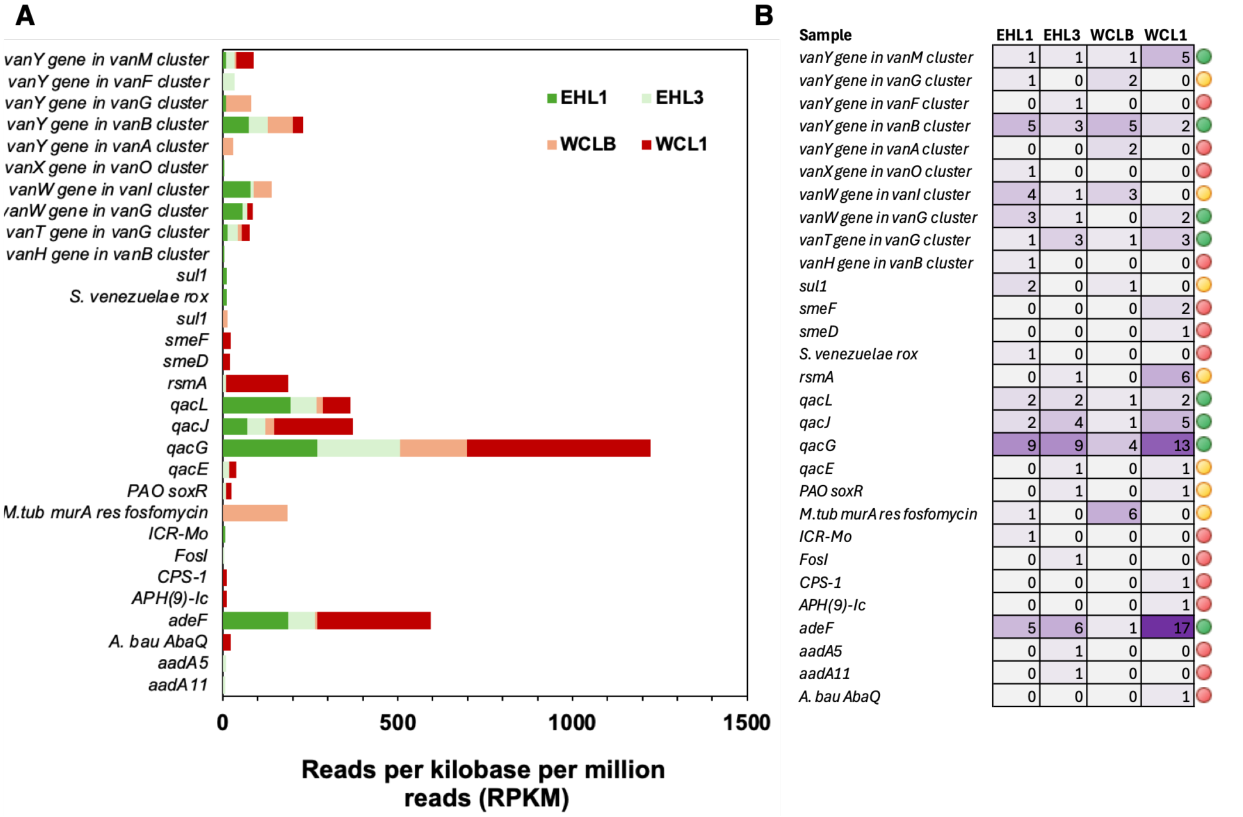

Fig. S6. Abundance of identified antimicrobial resistance genes (ARG) from the four metagenome swabs carrying contigs, quantified as reads per kilobase million (RPKM) and annotated using the CARD resistance genes identifier (RGI). Only hits with perfect and strict cut-off were considered. **B**. Gene frequency per sample. Green circles represent genes found in all four samples, yellow circles denote genes present in two or three samples, and red circles indicate genes found only in one sample. Note: In our analysis using RGI-CARD with strict and perfect hits only, no complete vancomycin resistance operons were detected in any MAG or contig.

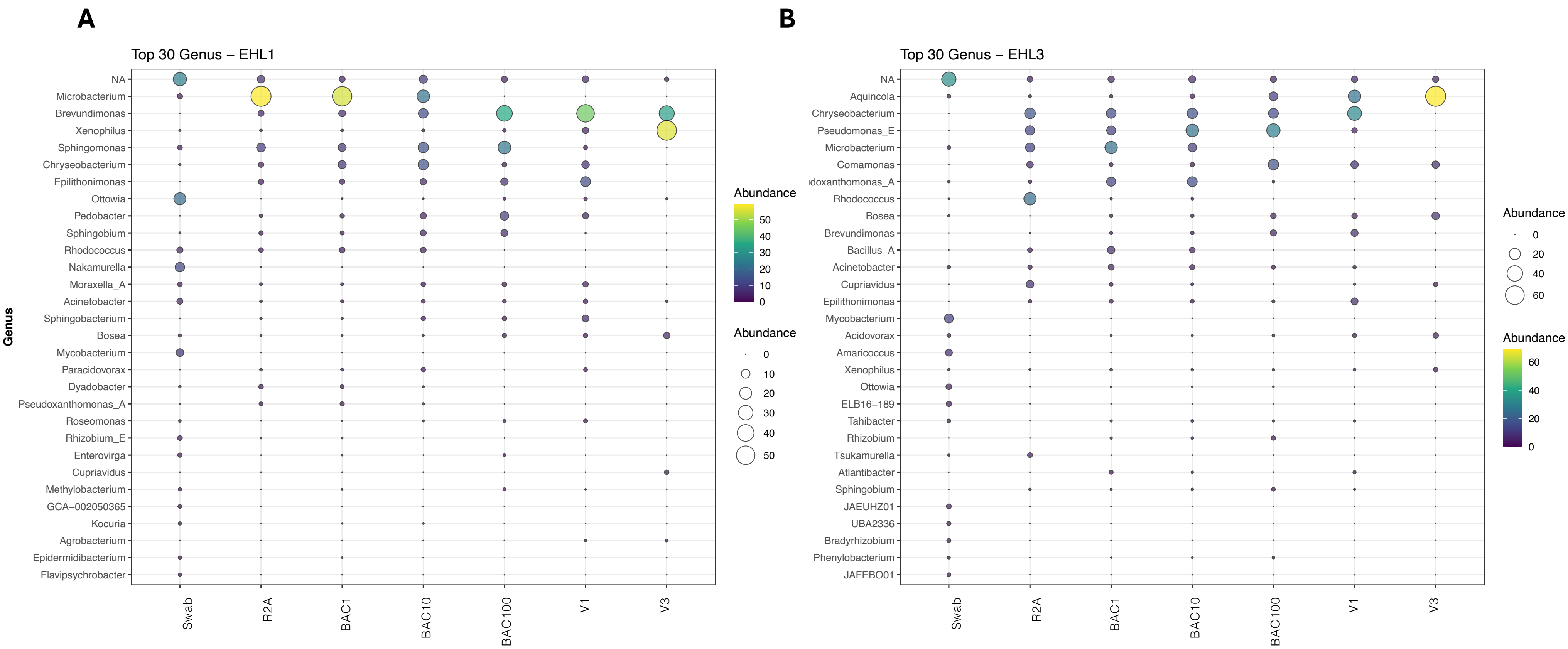

Fig. S7. Microbial community of EH biofilm sinks growing on disinfectant-containing agar media. First floor, L1 (A) and theird floor, L3 (B). Varying concentrations of BAC (C_{14} 50%, C_{12} 40%, C_{16} 10%) at 1 (BAC1), 10 (BAC10), and 100 (BAC100) mg/L, as well as commercial disinfectant Virex®Plus, tested at two dilutions: V1 (1:16,000 times), corresponding to a BAC concentration of 5.4 mg/L, and V3 (1:2,000 times - 8 times more diluted than recommended), corresponding to 43.5 mg/L BAC. The commercial disinfectant formulation included BAC (8.9%) and other QAC biocides: 6.67% octyl decyl dimethyl ammonium chloride, 2.67% dioctyl dimethyl ammonium chloride, and 4% dodecyl dimethyl ammonium chloride. Plates were incubated at room temperature for seven days.

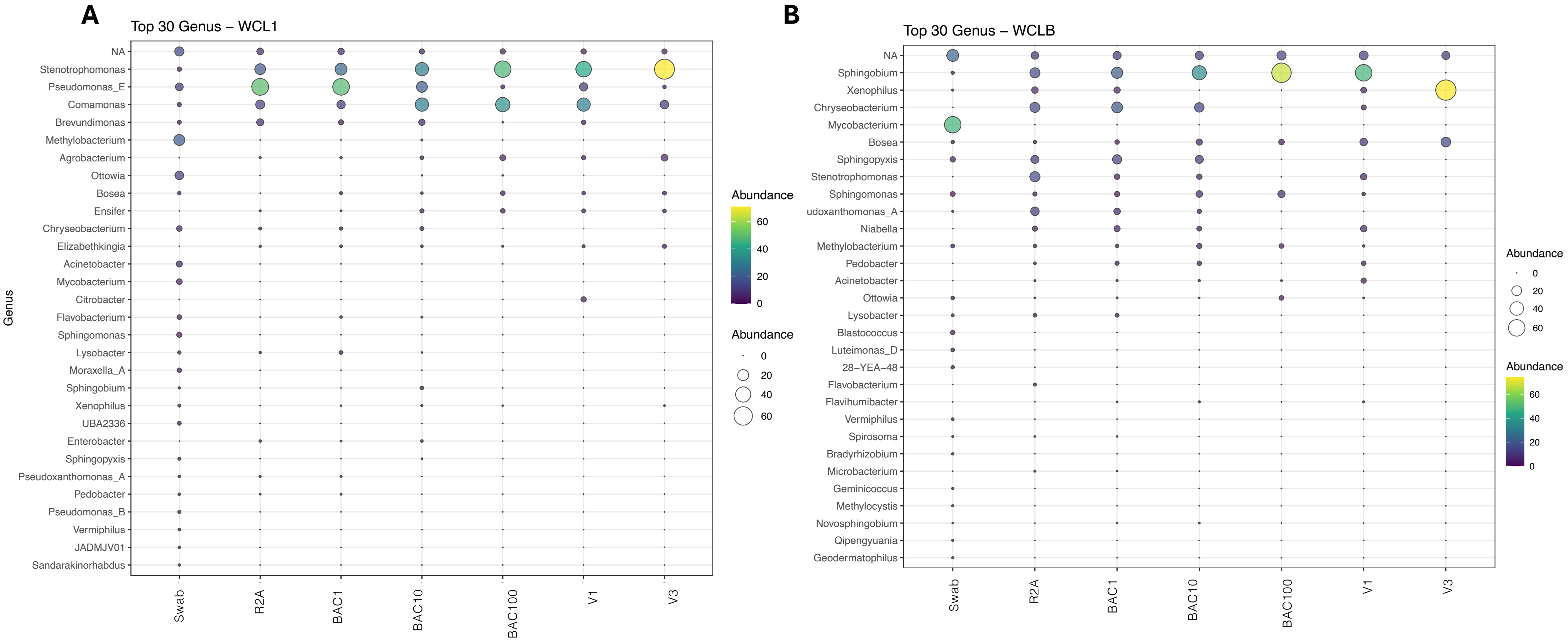

Fig. S8. Microbial community of WC biofilm sinks growing on disinfectant-containing agar media. First floor, L1 (A) and basement, LB (B). Varying concentrations of BAC (C_{14} 50%, C_{12} 40%, C_{16} 10%) at 1 (BAC1), 10 (BAC10), and 100 (BAC100) mg/L, as well as commercial disinfectant Virex®Plus, tested at two dilutions: V1 (1:16,000 times), corresponding to a BAC concentration of 5.4 mg/L, and V3 (1:2,000 times - 8 times more diluted than recommended), corresponding to 43.5 mg/L BAC. The commercial disinfectant formulation included BAC (8.9%) and other QAC biocides: 6.67% octyl decyl dimethyl ammonium chloride, 2.67% dioctyl dimethyl ammonium chloride, and 4% dodecyl dimethyl ammonium chloride. Plates were incubated at room temperature for seven days.

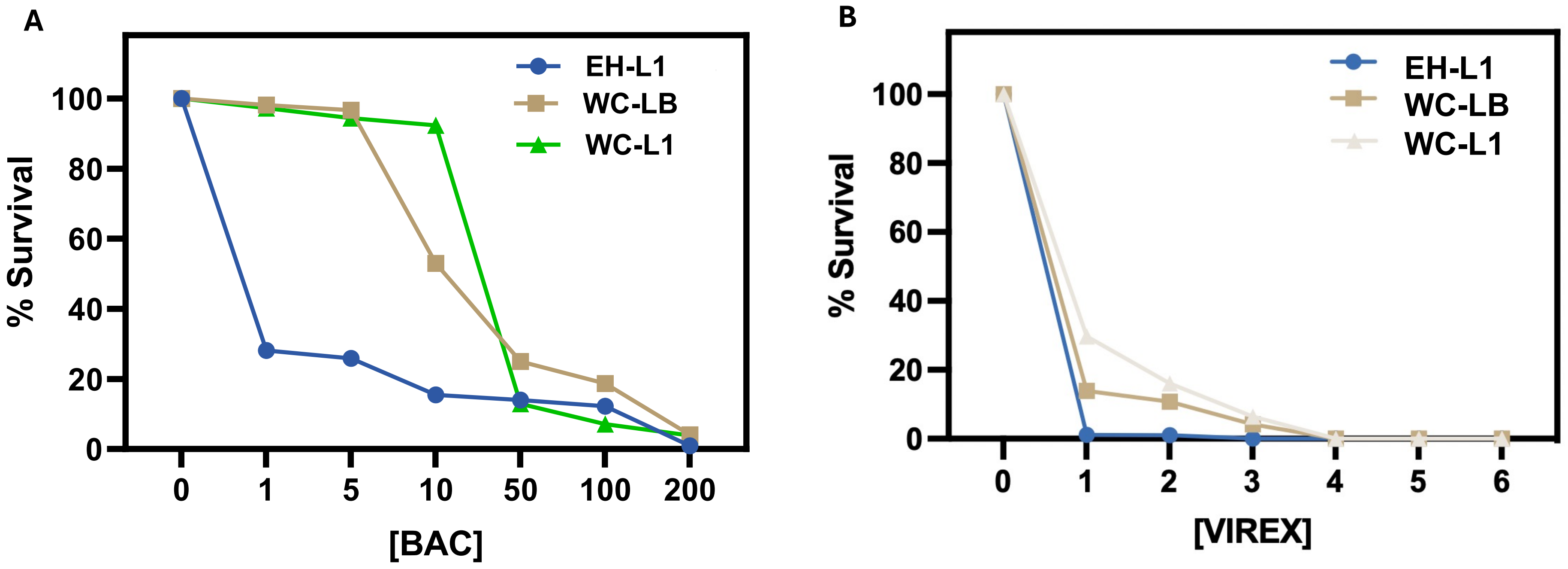

Fig. S9. Survival of bacteria exposed to increased concentration of disinfectants. **A**. Varying concentrations of BAC (C_{14} 50%, C_{12} 40%, C_{16} 10%) from 1 to 200 mg/L. **B**. Varying concentration of commercial disinfectant Virex®Plus, tested at six dilutions: V1 (1:16,000 times), corresponding to a BAC concentration of 5.4 mg/L, V2 (1:8,000 times; corresponding to 10.9 mg/L), V3 (1:2,000 times - 8 times more diluted than recommended), corresponding to 43.5 mg/L BAC; V4 (1:1024 times, 86.9 BAC mg/L); V5 (1:256 times, recommended dilution and equivalent to 347 mg BAC/L); and V6 (1:128 recommended dilution to remove viruses, equivalent to 695 mg BAC/L). The commercial disinfectant formulation included BAC (8.9%) and other QAC biocides: 6.67% octyl decyl dimethyl ammonium chloride, 2.67% dioctyl dimethyl ammonium chloride, and 4% dodecyl dimethyl ammonium chloride. Plates were incubated at room temperature for seven days.

Sample EH-L3 showed unexpected lower counts of CFU under R2A, non-exposed to BAC or Virex conditions.

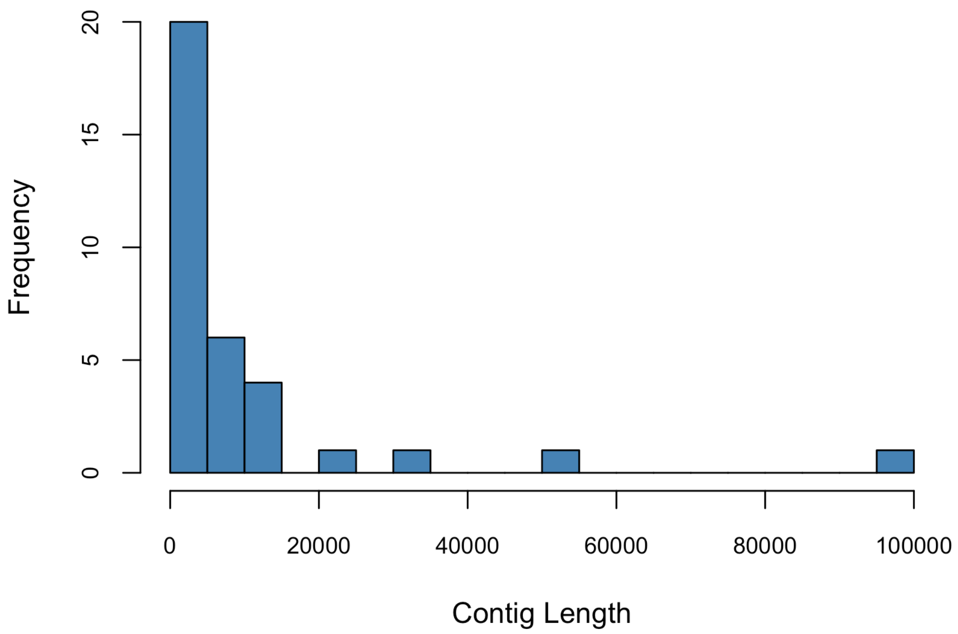

Fig. S10. Histogram showing the distribution of contig lengths carrying co-localized *qac* and plasmids genes. Majority of contigs (60%) are shorter than 5000 bp.
